# Preparation of Poly(ethylene glycol)@Polyurea Microcapsules Using Oil/Oil Emulsions and Their Application as Microreactors

**DOI:** 10.3390/polym13152566

**Published:** 2021-07-31

**Authors:** Ahmad Zarour, Suheir Omar, Raed Abu-Reziq

**Affiliations:** Casali Center of Applied Chemistry and The Center for Nanoscience and Nanotechnology, Institute of Chemistry, The Hebrew University of Jerusalem, Jerusalem 9190401, Israel; ahmad.zarour@mail.huji.ac.il (A.Z.); suheir.omar@mail.huji.ac.il (S.O.)

**Keywords:** non-aqueous interfacial polymerization, polyurea microcapsules, poly(ethylene glycol), oil-in-oil emulsions, hydrogenation

## Abstract

The development process of catalytic core/shell microreactors, possessing a poly(ethylene glycol) (PEG) core and a polyurea (PU) shell, by implementing an emulsion-templated non-aqueous encapsulation method, is presented. The microreactors’ fabrication process begins with an emulsification process utilizing an oil-in-oil (o/o) emulsion of PEG-in-heptane, stabilized by a polymeric surfactant. Next, a reaction between a poly(ethylene imine) (PEI) and a toluene-2,4-diisocyanate (TDI) takes place at the boundary of the emulsion droplets, resulting in the creation of a PU shell through an interfacial polymerization (IFP) process. The microreactors were loaded with palladium nanoparticles (NPs) and were utilized for the hydrogenation of alkenes and alkynes. Importantly, it was found that PEG has a positive effect on the catalytic performance of the developed microreactors. Interestingly, besides being an efficient green reaction medium, PEG plays two crucial roles: first, it reduces the palladium ions to palladium NPs; thus, it avoids the unnecessary use of additional reducing agents. Second, it stabilizes the palladium NPs and prevents their aggregation, allowing the formation of highly reactive palladium NPs. Strikingly, in one sense, the suggested system affords highly reactive semi-homogeneous catalysis, whereas in another sense, it enables the facile, rapid, and inexpensive recovery of the catalytic microreactor by simple centrifugation. The durable microreactors exhibit excellent activity and were recycled nine times without any loss in their reactivity.

## 1. Introduction

The pursuit of a sustainable future has become the driving force for today’s science, where pioneering approaches toward greener processes are continually being developed. In this regard, the design of efficient and facile catalytic systems is highly desired [1,2]. Although the homogeneous route of catalysis is endowed with high reactivity and selectivity [3,4], most of the industrial catalytic processes rely on the heterogeneous counterpart owing to significant recovery and cost concerns [5,6]. Many efforts have been devoted to bridging the gap between the two routes and to utilizing their advantages; in this regard, numerous methods have been developed. Among others, this includes the use of Pickering emulsion systems [7,8], anchored single-atom catalysts [9], mesoscale nanostructures [10], and tunable solvents [11].

The use of metal nanoparticles constitutes another powerful tool for combining the advantages of both disciplines because of metal nanoparticles’ high surface area [12,13]. However, despite their great reactivity and selectivity, metal nanoparticles require additional immobilization techniques to avoid their aggregation, and usually involve the use of different supports to enable catalyst recovery. The uses of organic polymer supports [14,15,16], dendrimers [17,18,19], supported ionic liquid phase (SILP) catalysts [20,21,22], magnetic nanoparticles (MNPs) [23,24,25], and inorganic supports [26,27,28] are just a few examples of many immobilization techniques available for metal nanoparticles. However, along with leaching problems, these methods generally involve multiple synthetic steps and pre-functionalization processes, which make the development of new catalysts difficult, expensive, and cumbersome. Moreover, generally, their reactivity and selectivity are less efficient in comparison with the pure homogeneous catalysts.

Recently, catalysis within microenvironmental entities, called in brief microreactors, has been proven to be efficient at least as the pure homogeneous route. The remarkably enhanced catalytic performance refers to the local high concentrations of reagents within the dispersed microreactors, a fact that dramatically increases the reaction rate constants [29,30,31,32,33,34]. Accordingly, microcapsules (MCs) might serve as a useful tool for inducing such microenvironmental catalytic conditions [35,36,37,38]. Microencapsulation involves the process of confining various types of ingredients for diverse purposes, such as the controlled release of pesticides in agriculture [39,40,41], the release of drugs in pharmaceutical applications [42,43,44], the masking of odors and tastes in the food industry [45,46,47], and the protection and recycling of catalysts [48,49,50,51,52].

Interfacial polymerization (IFP) is a well-known chemical method for preparing MCs [53,54,55,56]. This process is based on a polycondensation reaction between two highly reactive monomers; it takes place at the boundary of two immiscible phases, and each phase contains one of the two monomers. Polyurea (PU) is the most common polymer prepared via IFP for the synthesis of MCs; it has been thoroughly investigated and applied in different fields [57,58,59,60,61,62]. PUs are generated from the reaction between amine and isocyanate monomers. These MCs are considered valuable owing to their unique features, such as low Tg, rubbery characteristics, as well as their chemical [61] and mechanical [60] stability and biocompatibility, [63] which make them an ideal material for holding liquid in their core even for in vitro applications. In addition to the encapsulation of ionic liquids, which was demonstrated by our group [64], phase change materials [65] and more common organic solvents, such as xylene [66], were found to serve as an efficient platform for implementing PU MCs. The encapsulation process in the latter examples was preceded by an oil-in-water (o/w) emulsification process. Furthermore, water-in-oil (w/o) emulsion-templated PU MCs have been reported as well [67]. However, these two emulsification strategies (o/w and w/o) cannot provide an adequate solution in the case of moisture-sensitive ingredients, including catalysts. Thus, the development of a non-aqueous process, such as the use of oil-in-oil (o/o) emulsions, is of the utmost importance [68,69,70,71,72]. Although PU MCs and o/o emulsions themselves are well-established topics, their combination has barely been studied [73,74,75,76,77]. In addition, palladium NPs have been successfully supported on different catalytic systems, including magnetic carbon-coated cobalt nanobeads [78], silicon nanowire arrays [79], and cotton and filter paper [80], and were applied in the hydrogenation of alkenes. Moreover, palladium NPs were successfully encapsulated within PU MCs based on oil-in-water [81,82,83] and water-in-oil [84] emulsions.

Herein, we present the first palladium-based PU microreactor prepared via a non-aqueous process that utilizes an o/o emulsion. A low-molecular-weight poly (ethylene glycol) (PEG) was successfully encapsulated, for the first time, within a PU shell. PEG represents a green reaction medium owing to its low vapor pressure and it is considered as a GRAS (generally recognized as safe) material [85,86]. In addition to its acting as a green medium for organic transformations, PEG was also found to play two additional roles that have a direct positive impact on the catalytic performance. First, it acts as a good stabilizing agent for palladium NPs, which is in line with the results obtained in other works [87,88,89,90]. Thus, it prevents the aggregation of palladium NPs and increases their reactivity. Second, it acts as a reducing agent, avoiding the need for additional reducing agents to activate the palladium NPs. Recently, PEG-based microcapsules were developed and applied for energy storage [91,92] or drug formulation [93,94,95] and for creating unique multilayer polymeric shells with complex structures [96].

## 2. Materials and Methods

### 2.1. Materials

The organic solvents heptane, toluene, xylene, and cyclohexane were purchased from Romical, along with chemicals and laboratory equipment. Poly(1-vinylpyrrolidone)-graft-(1-hexadecene) co-polymer (containing 80% by weight of C_16_ α-olefin and 20% by weight of polymerized vinylpyrrolidone) (Agrimer AL-22) was purchased from International Specialty Products (ISP). Bis-poly(ethylene glycol)/poly(propylene glycol)-14/14 dimethicone (ABIL EM 97) was purchased from Evonik. Poly(ethylene glycol) 200, sodium tetrachloropalladate (Na_2_PdCl_4_), dioctyl sulfosuccinate sodium salt (AOT), sorbitan monooleate (span 80), poly(ethylene glycol) oleyl ether (brij 92v), branched poly(ethylene amine) 800, 2,2′(ethylenedioxy)bis(ethylamine), hexamethylenediamine (HMDA), and diethylenetriamine (DETA) were purchased from Sigma Aldrich-Merck. Polymethylene polyphenyl isocyanate (PAPI 27) was contributed by FMC Corporation. Toluene-2,4-diisocyanate (TDI) was purchased from Acros Organics. All substrates were purchased either from Alfa Aesar (Tewksbury, MA, USA) or Sigma Aldrich-Merck (Budapest, Hungary).

### 2.2. Instrumentation

The o/o emulsion containing 0.01% Rhodamine B in PEG200 was analyzed by a fluorescence microscope (Carl Zeiss, Axio Vision, Mátészalka, Hungary). The catalytic microcapsules were initially examined using a high-resolution scanning electron microscope (HR SEM) Sirion from FEI equipped with a Shottky-type field emission source and a secondary electron (SE) detector at 5 kV. Focused ion beam (FIB-SEM), using a FEI Helios nanolab 460S1 instrument, was used to investigate the inner and outer morphology of the microcapsules and to evaluate whether core–shell or matrix structures were obtained. Moreover, it was utilized for the mapping and EDXS (energy dispersive X-ray spectroscopy) analyses. 1H-NMR measurements were performed using a Bruker DRX-400 spectrometer to determine conversion and selectivity. Emulsifications were performed using the Kinematica Polytron homogenizer PT-6100 equipped with dispersing aggregate 3030/4EC. IR was recorded with KBr pellets at room temperature in transmission mode on a PerkinElmer 65 FTIR spectrometer to determine the chemical composition of the microcapsules. In addition, thermogravimetric analysis (TGA) was performed using a Mettler Toledo TG 50 analyzer at a temperature range of 25–700 °C and at a heating rate of 10 °C min^−1^ under N_2_ atmosphere. Scanning transmission electron microscopy (STEM) and electron diffraction spectroscopy (EDS) were performed with (S)TEM Tecnai F20 G2 (FEI Company, Hillsboro, OR, USA) operated at 200 kV.

### 2.3. The Procedure for Preparing the Pd_NPs_/PEG_200_ Polar Phase

In a vial of 10 mL, 100 mg (0.287 mmol) of Na_2_PdCl_4_ was dissolved and sonicated in 4.66 g of PEG_200_ for one hour. At this stage, the mixture’s color changes from light red to dark red and then to black, indicating that the palladium was reduced. Then, 0.34 g of an amine was added and the mixture was allowed to sonicate for one more hour.

### 2.4. General Procedure for Preparing the Pd_NPs_/PEG_200_@PU Microreactors

In a 100 mL beaker, 2.00 g of surfactant was dissolved in 13.40 g of heptane. The solution was homogenized at 10,000 rpm for 30 s. Then, the Pd_NPs_/PEG_200_ polar phase was added and the homogenization proceeded for another two minutes. Afterward, a mixture of 0.6 g of an isocyanate dissolved in 4 g of xylene was added dropwise. The emulsion was allowed to stir at 500 rpm on a stirring plate for 4 h at room temperature. The resulting catalytic microreactors were separated by centrifugation and washed three times with heptane. The Pd_NPs_/PEG_200_@PU MCs were then dispersed in heptane until the weight of the whole dispersion was 20.00 g.

### 2.5. General Procedure for the Hydrogenation Reaction

In a 25 mL glass-lined autoclave equipped with a magnetic stirring bar, 1 g of the catalytic dispersion (0.047 mmol of Pd per 1 g of dispersion) was added to 2 mL of heptane. Then, a suitable amount of substrate was added relative to the substrate/catalyst (S/C) ratio. The autoclave was purged three times with H_2_ and then pressurized to 100 psi of H_2_. The reactions were carried out at room temperature for 2.5 h. Finally, the MCs were separated from the reaction mixture by centrifugation, washed two times with heptane, redispersed in 3 mL of heptane, and then used for the next catalytic cycle. The reaction content was examined using ^1^H-NMR spectroscopy in order to determine the conversion and selectivity.

## 3. Results and Discussion

### 3.1. Synthesis and Optimization of the PEG_200_@PU MCs

**Scheme 1:**
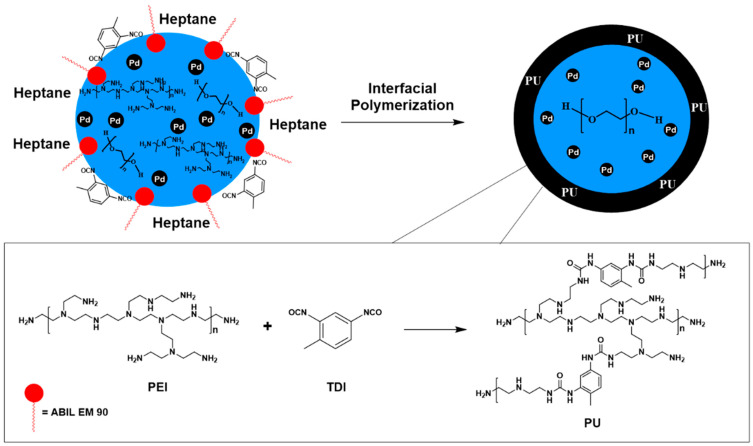
Schematic illustration of the Pd_NPs_/PEG_200_@PU preparation procedure.

The synthesis of the polyurea microcapsules (PU MCs) is based on an emulsion-templated non-aqueous encapsulation process. The preparation procedure begins with an emulsification process consisting of two immiscible oil phases: (1) the polar dispersed phase consists of branched poly(ethylene imine)_800_ (PEI_800_) and Na_2_PdCl_4_ dissolved in poly(ethylene glycol)_200_ (PEG_200_), and the PEG_200_ rapidly reduces the palladium (II) ions to palladium (0) nanoparticles (NPs); and (2) a polar continuous phase of heptane containing the polymeric surfactant ABIL EM 90. Then, a toluene-2,4-diisocyanate (TDI) dissolved in xylene was slowly added while the homogenization process was running. Finally, an interfacial polymerization process at the boundary of the emulsion droplets between the PEI and the TDI is executed, leading to the creation of a PU MC possessing a core/shell structure owing to the insolubility of the PU in PEG (Scheme 1).

The selection of these materials was determined after conducting a series of optimization experiments in which other continuous phases (cyclohexane and toluene), surfactants (Agrimer AL-22, AOT, span 80, and brij 92v), amines (2,2′(ethylenedioxy)bis(ethylamine), HMDA, and DETA) and isocyanate (PAPI 27) were examined. Briefly, excluding the polymeric surfactants, ABIL EM 90, and Agrimer AL-22 (Figure S1), none of the surfactants were able to stabilize the o/o emulsion; the stabilization of such emulsions is not obvious and usually requires the involvement of multi-armed polymeric surfactants rather than small ones; the former settle irreversibly at the interface of the droplets, as their departure from the interface requires that all the surfactants’ arms leave the interface concomitantly, which is statistically less possible. Moreover, whereas the interfacial polymerization of the PEI/TDI and DETA/TDI pairs fabricates MCs, the 2,2′(ethylenedioxy)bis(ethylamine)/PAPI 27 and HMDA/PAPI 27 pairs result in the formation of nanocapsules (Table S1). However, the latter two suffer from aggregation problems, whereas in the case of the DETA/TDI pair, the encapsulation of PEG_200_ is not attainable. In the case of cyclohexane only, the DETA/TDI pair affords MCs when Agrimer AL-22 was utilized as the emulsion stabilizer (Figure S2); however, as already mentioned, the encapsulation of PEG_200_ with this pair is not viable. For toluene, well-defined MCs were not obtained with either of the pairs.

### 3.2. Characterization of the Pd_NPs_/PEG_200_@PU MCs

**Figure 1:**
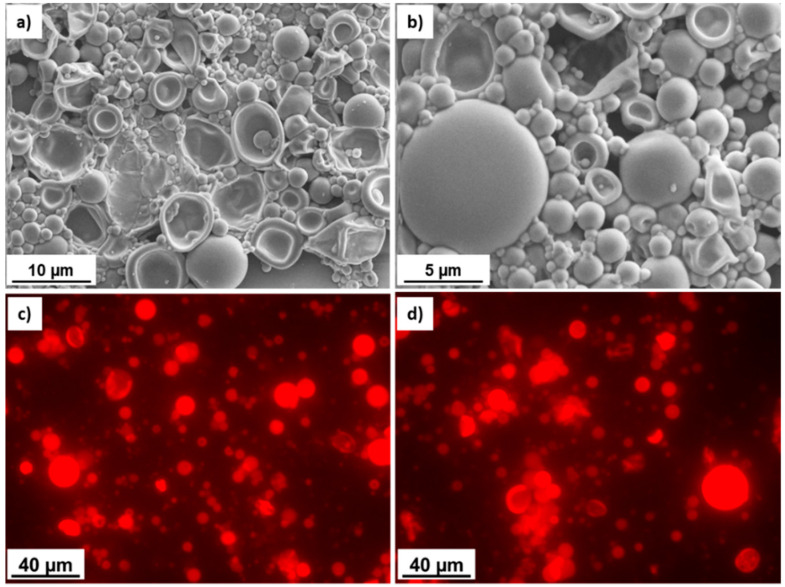
(**a**,**b**) Scanning electron microscopy (SEM) and (**c**,**d**) fluorescent microscopy images of PEG_200_@PU microcapsules (MCs).

First, the catalytic system was investigated before the penetration of the palladium. Scanning electron microscopy (SEM) was employed for determining the morphological structure of our system (Figure 1a,b). Smoothed spherical surfaces and some pressed capsules, caused by the high vacuum applied in this analysis, were obtained. As can be easily noted, a polydispersed system with sizes ranging from 200 nm to 15 µm was formed, which is a typical feature of macroemulsion systems. The measurement of the size of the microcapsules dispersed in isopropyl alcohol by laser diffraction size analyzer indicated an average size of 12.48 µm (d_0.5_ = 12.48 micron, Figure S3). Moreover, fluorescent microscopy images indicate the presence of the liquid PEG_200_, accompanied by 0.01 wt.% of Rhodamine B dye, within the PU MCs (Figure 1c,d).

**Figure 2:**
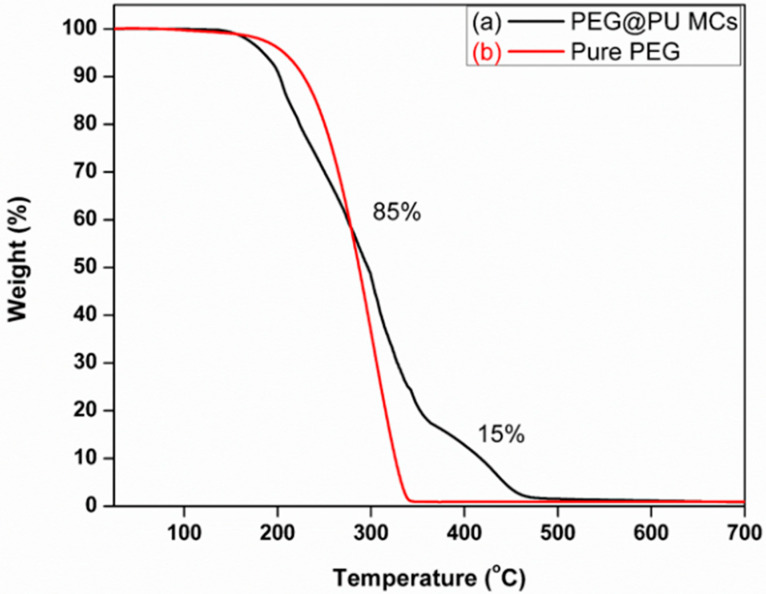
Thermogravimetric analysis (TGA) curves of (**a**) PEG_200_@PU MCs and (**b**) pure PEG200.

Thermogravimetric analysis (TGA) indicates the existence of two weight loss stages (Figure 2). The first weight loss is associated with the decomposition of the encapsulated PEG_200_. This weight loss occurs almost at the same range of temperatures at which pure PEG_200_ decomposes (200–300 °C), and constitutes 85% of the sample’s weight, which is in agreement with the calculated wt.% of PEG_200_ from the total amount of MCs (87%). The second weight loss (15%), centered at 400 ℃, apparently is associated with the thermal decomposition of the PU shell, which, according to the theoretical calculations, stands at 13%. Moreover, the MCs are thermally stable up to 200 °C; this makes them applicable even when elevated temperatures are needed.

**Figure 3:**
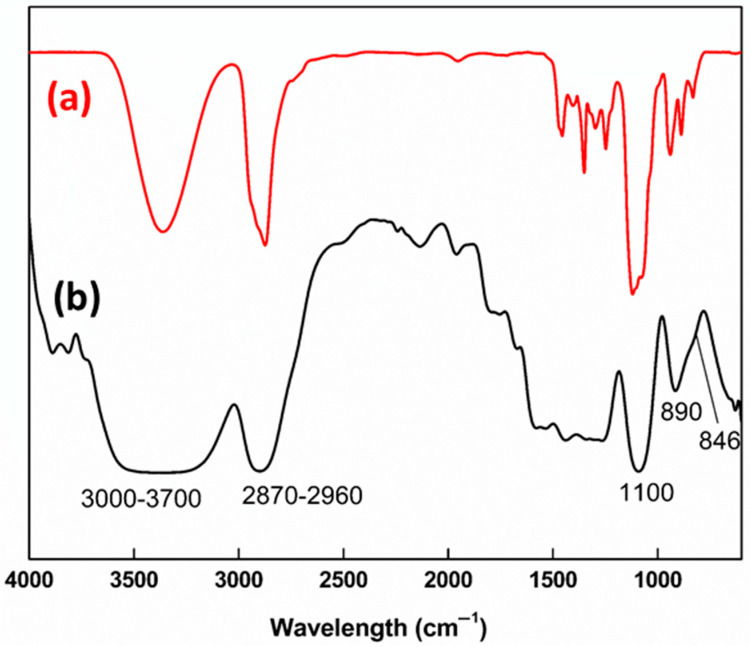
Fourier-transform IR (FT-IR) spectra of (**a**) pure PEG200 and (**b**) PEG_200_@PU MCs.

Fourier-transform IR (FT-IR) was further employed to confirm the chemical composition in the system. The results presented in Figure 3 revealed that polyurea polymerization was achieved. The absence peak band at ~2270 cm^−1^ confirms the total polymerization of the isocyanate monomer (TDI), which apparently reacted completely with the branched PEI_800_ to form the polyurea shell. The absorption bands at 846 cm^−1^ (CH_2_ rocking), 890 cm^−1^ (C-OH bending), 1100 cm^−1^ (C-O stretching), and 2870–2960 cm^−1^ refer to PEG_200_, whereas the wide absorption band from 3000 to 3700 cm^−1^ is attributed to the N-H and O-H stretching vibrations of polyurea and PEG.

**Figure 4:**
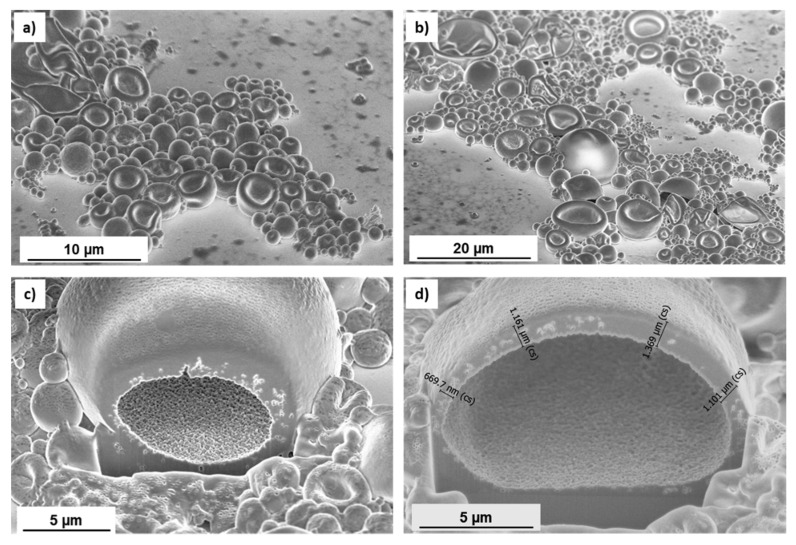
Focused ion beam (FIB)-SEM images of the PdNPs/PEG200@PU MCs before etching process (**a**,**b**) and after etching (**c**,**d**).

After confirming the feasibility for the formation of the MCs, they were loaded with palladium NPs and characterized by different methods. The focused ion beam (FIB-SEM) images indicate that the MCs maintain their morphology and their spherical structure after the penetration of the palladium (Figure 4a,b). Moreover, a cutting process confirms the existence of a core/shell structure with a shell thickness of ~1 µm (Figure 4c,d); however, the thickness varied, depending on the MC size. The presence of the carbon, nitrogen, and oxygen elements was further confirmed by conducting mapping and EDXS (energy dispersive X-ray spectroscopy) analyses, for the cut MC and the complete MCs (Figures S4 and S5).

**Figure 5:**
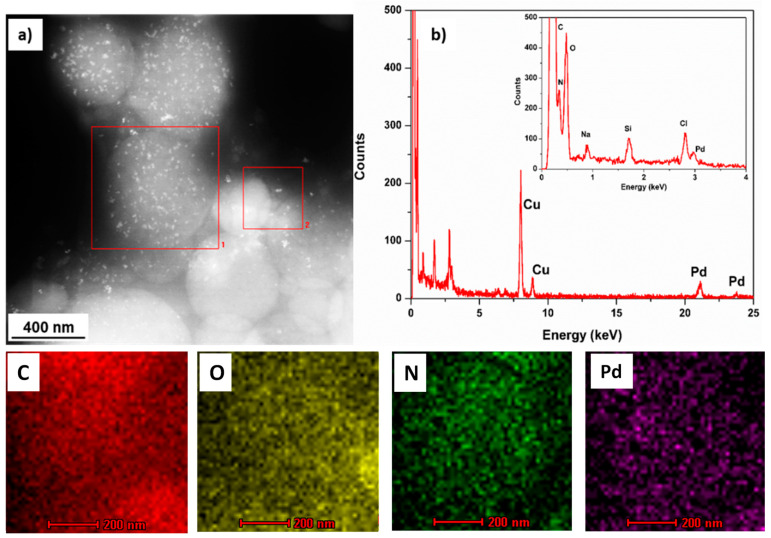
STEM mode applied for the (**a**) mapping and (**b**) energy dispersive X-ray spectroscopy (EDXS) analyses of the Pd_NPs_/PEG_200_@PU MCs.

However, although the EDXS analysis confirms the existence of palladium, the mapping measurement was not sensitive enough to detect the presence of palladium. In this regard, we carried out the mapping measurement in scanning transmission electron microscopy (STEM) mode (Figure 5a). Moreover, the figure reveals that the palladium NPs were successfully encapsulated within the MCs. The presence of palladium was also verified by EDXS analysis (Figure 5b). Besides the already known positive stabilization effect of the PEG [87,88,89,90], the very small palladium NPs are further stabilized by the nitrogen atoms of the branched PEI_800_ and by the microencapsulation process itself; the latter constructs a concrete barrier between the palladium NPs and constitutes a hurdle to the possibility of high constrictions of palladium NPs, which eventually will lead to aggregation processes and, subsequently, to a drop in the catalytic activity.

### 3.3. Evaluation of the Catalytic Performance

The catalytic performance of the Pd_NPs_/PEG_200_@PU microreactor was tested in the hydrogenation of various alkenes and alkynes. As shown in Table 1, the catalyst exhibits highly desirable activity in the hydrogenation of alkenes. Fully saturated alkanes could be generated smoothly under mild conditions (Table 1, entries 1–6). The catalyst’s activity was also tested in the hydrogenation of alkynes; aromatic terminal alkynes were fully converted to the corresponding fully hydrogenated products (Table 1, entries 7–9). Interestingly, *para*-substrates, such as 4-vinylanisole and 4-methylstyrene, exhibit a slightly better catalytic performance compared with *meta*-substrates, such as 3-methylstyrene and 3-chlorostyrene. Nevertheless, such a finding requires further kinetic experiments and an in-depth investigative study of the microreactor structure’s porosity. In addition, diphenylacetylene, which was also fully converted, exhibits exceptional behavioral patterns with a moderate selectivity of 61% towards the *cis*-stilbene and 39% towards the fully hydrogenated bibenzyl. This partial selectivity could be attributed to the steric hindrance of the *cis*-stilbene formed initially, which can slow its hydrogenation within the Pd/PEG@polyurea microreactor (Table 1, entry 10). An excellent reactivity of our catalyst was also achieved when a substrate/catalyst ratio of 5000 was applied in the hydrogenation of styrene (Table 1, entry 11).

Furthermore, the catalytic performance of the developed microreactor was compared with a completely homogeneous system. In this regard, the hydrogenation of styrene was used as a model reaction. Strikingly, the Pd_NPs_/PEG_200_@PU microreactor exhibits catalytic supremacy over the pure homogeneous catalyst under the same conditions; the homogeneous catalyst reached 28% in PEG_200_, whereas the microreactor reached 100% conversion. These results indicate that the PEG stabilization does not ensure high catalytic reactivity; however, performing the reaction within a microenvironmental entity guarantees a high local concentration of interactions between the catalyst and the substrates, leading to an improved catalytic performance and highly efficient processes.

**Figure 6:**
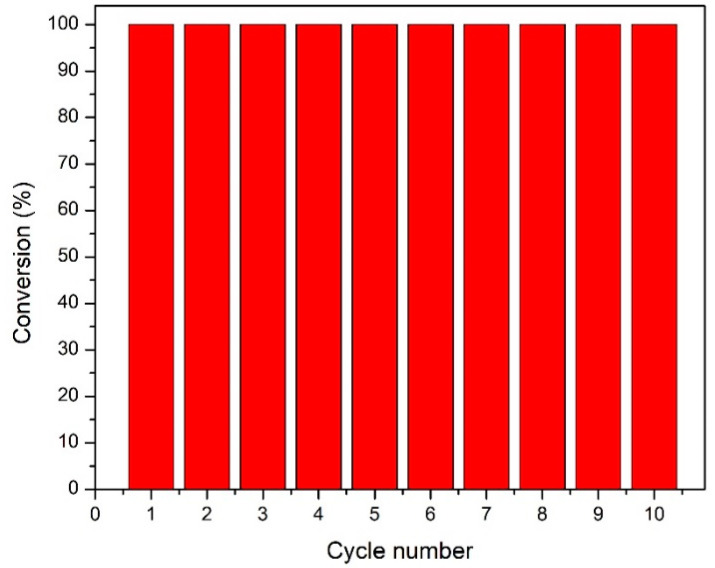
Recyclability of the Pd_NPs_/PEG_200_@PU microreactor in the hydrogenation reaction of styrene (reaction conditions: 0.2 mol% of Pd (substrate/Pd ratio = 500), 2.5 h, r.t., 100 psi of H_2_).

Finally, the recyclability of our catalyst was examined. The catalyst was recycled nine times without showing any loss of its activity, as seen in Figure 6. The catalyst was easily separated by centrifugation, washed, and reused for the next cycle.

## 4. Conclusions

With our continuous intent to bridge the gap between the homogeneous and heterogeneous routes, highly reactive Pd_NPs_/PEG_200_@PU microreactors were successfully developed. The fabrication process is based on implementing an o/o emulsion-templated non-aqueous microencapsulation, through the interfacial polymerization of PEI_800_ and TDI. These microreactors were well characterized and utilized in the hydrogenation of aromatic alkenes and alkynes, exhibiting extraordinary reactivity. The catalytic supremacy results from the very small and efficient palladium NPs; such a stabilization is achievable owing to the presence of PEG, which, to the best of our knowledge, was not encapsulated previously within a PU shell, surely not by a non-aqueous route. Not less important is the existence of the microenvironmental entity, which allows high local concentrations of interactions between the catalyst and substrates. Finally, the robust PU shell maintains the spherical structure of the microreactor and enables its facile recovery and recyclability.

## Figures and Tables

**Table 1:** The catalytic performance of the Pd_NPs_/PEG_200_@PU microreactor in the hydrogenation reaction.

Entry	Substrate	Conversion (%) ^a^	Entry	Substrate	Conversion (%) ^a^
1	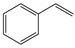	100	7	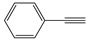	100
2	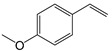	100	8	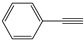	100
3	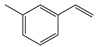	96	9	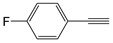	100
4	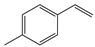	100	10 ^b^	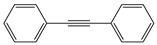	100
5	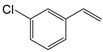	92	11 ^c^	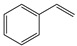	100
6	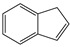	100			

Reaction conditions: 0.2 mol% of Pd (substrate/Pd ratio = 500), 2.5 h, r.t., 100 psi of H_2_. ^a^ Determined by ^1^HNMR. ^b^ 61% *cis*-stilbene and 39% bibenzyl. ^c^ 0.02 mol% of Pd (substrate/Pd ratio = 5000).

## Data Availability

The data presented in this study are available on request from the corresponding author.

## Supplementary Materials

The following are available online at https://www.mdpi.com/article/10.3390/polym13152566/s1, Figure S1: SEM images of PEG@PU microcapsules prepared using different surfactants; Table S1: PEG_200_@PU microcapsules obtained using different amines and isocyanate monomers; Figure S2: SEM image of PEG_200_@PU microcapsules obtained using PEG_200_-in-cyclohexane emulsions; Figure S3: FIB-SEM images of Pd_nano_/PEG_200_@PU microcapsules.
