# Supplementary material for: Preparation of Poly(ethylene glycol)@Polyurea Microcapsules Using Oil/Oil Emulsions and Their Application as Microreactors
Source: Polymers (Basel). 2021 Jul 31;13(15):2566. doi: 10.3390/polym13152566 (PMC8348332; doi:10.3390/polym13152566)
Supplement: Supplementary file 1 [file polymers-13-02566-s001.zip › polymers-1320411-supplementary.pdf]

Supporting Information

Preparation of Poly(ethylene glycol)@Polyurea Microcapsules Using Oil/Oil emulsions and their Application as Microreactors

Ahmad Zarour[1], Suheir Omar[1] and Raed Abu-Reziq[1*]

Institute of Chemistry, Casali Center of Applied Chemistry and the Center for Nanoscience and Nanotechnology, The Hebrew University of Jerusalem, Jerusalem 9190401, Israel.

* Correspondence: Raed.Abu-Reziq@mail.huji.ac.il; Tel.: +972-2-6586097; Fax: +972-2-6585469

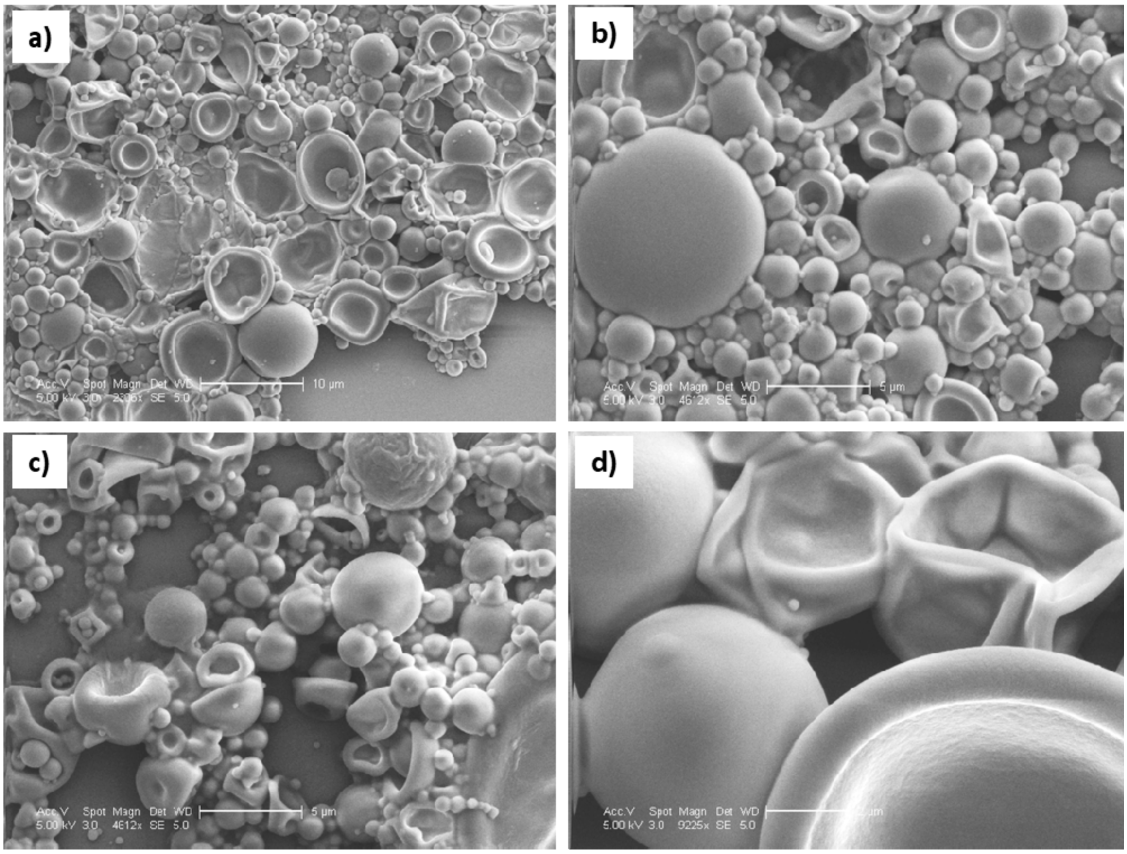

Figure S1. PEG_{200}@PU MCs obtained with (a, b) ABIL EM 90 and (c, d) Agrimer AL-22 (The continuous phase is heptane, the amine is PEI and the isocyanate is TDI).

Table S1. PEG_{200}@PU MCs obtained by different IFP pairs of amines and isocyanates (the continuous phase is heptane and the surfactant is ABIL EM 90).

**Interfacial polymerization pair**	**SEM images**
PEI/TDI	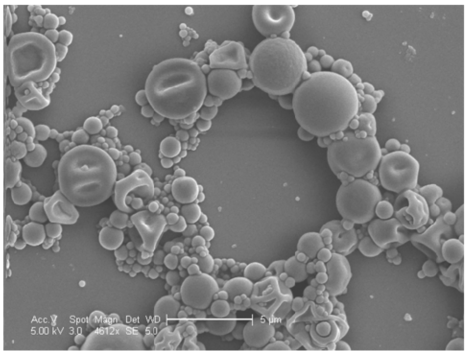 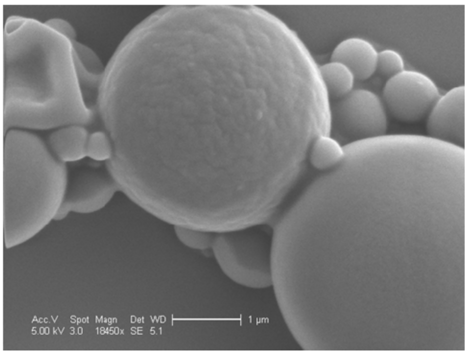
DETA/TDI	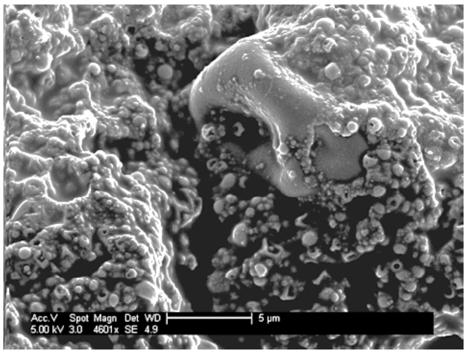 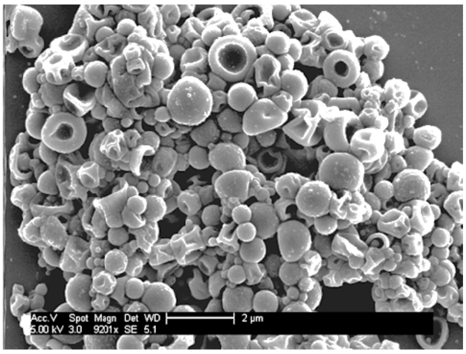

2,2′(ethylenedioxy)bis(ethylamine)/ PAPI 27	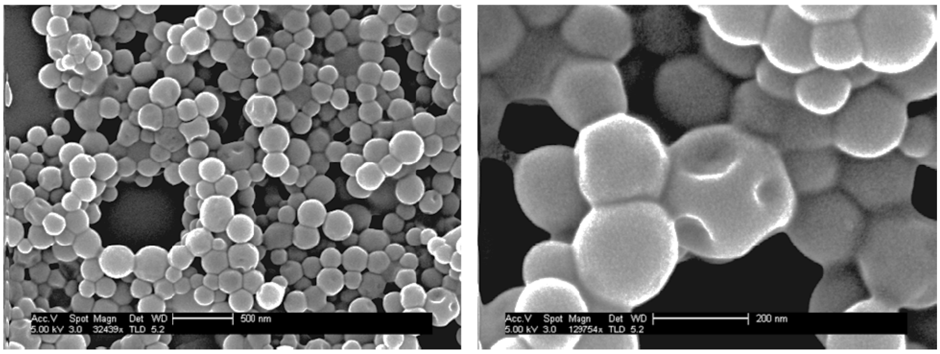
HMDA/ PAPI 27	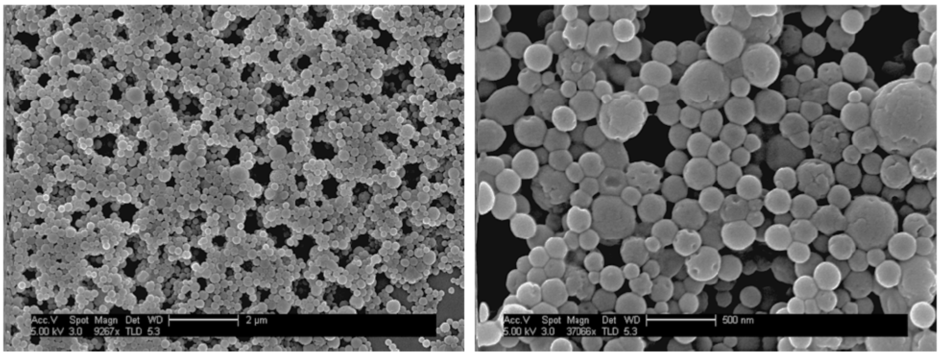

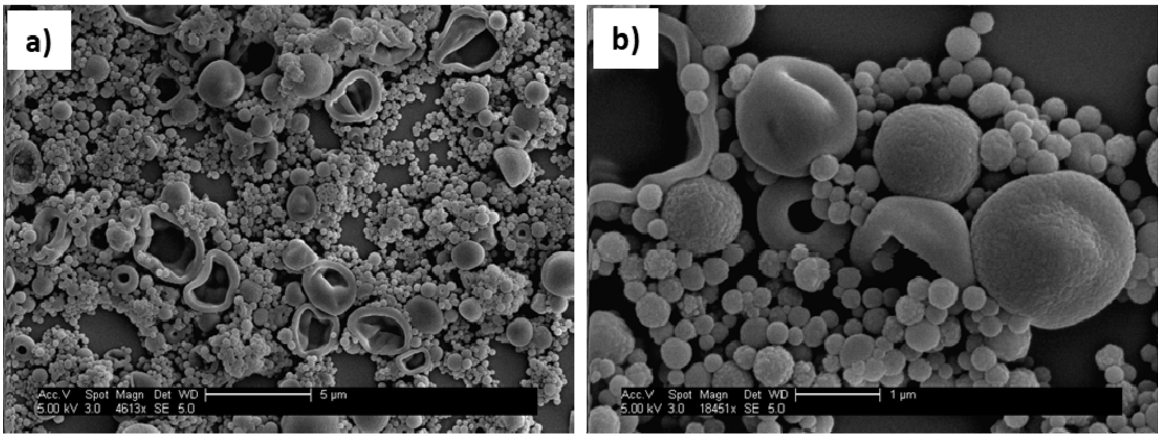

Figure S2. (a, b) PEG_{200}@PU MCs obtained with cyclohexane (The surfactant is Agrimer AL-22, the amine is DETA and the isocyanate is TDI).

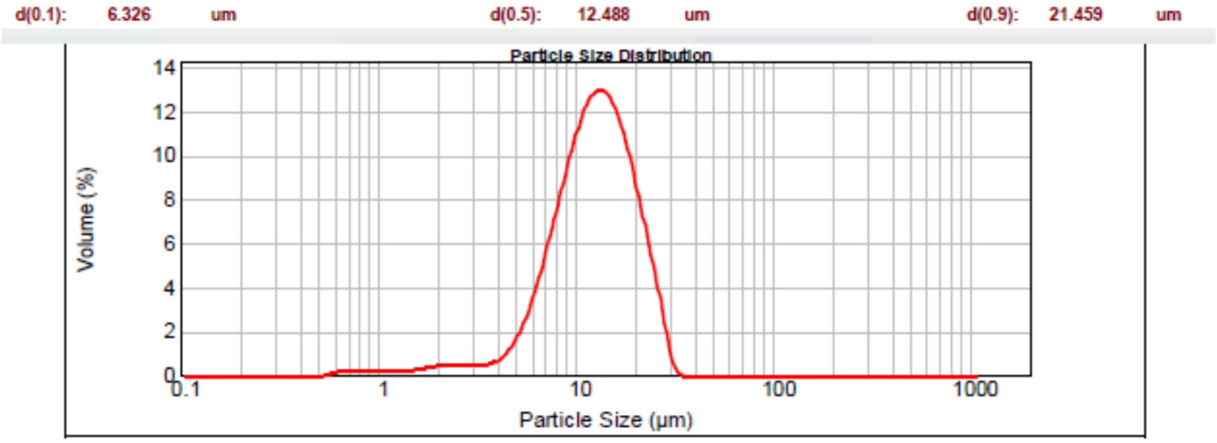

Figure S3. Size distribution of the PEG_{200}@PU microcapsules dispersed in isopropyl alcohol as measured by laser diffraction size analyzer

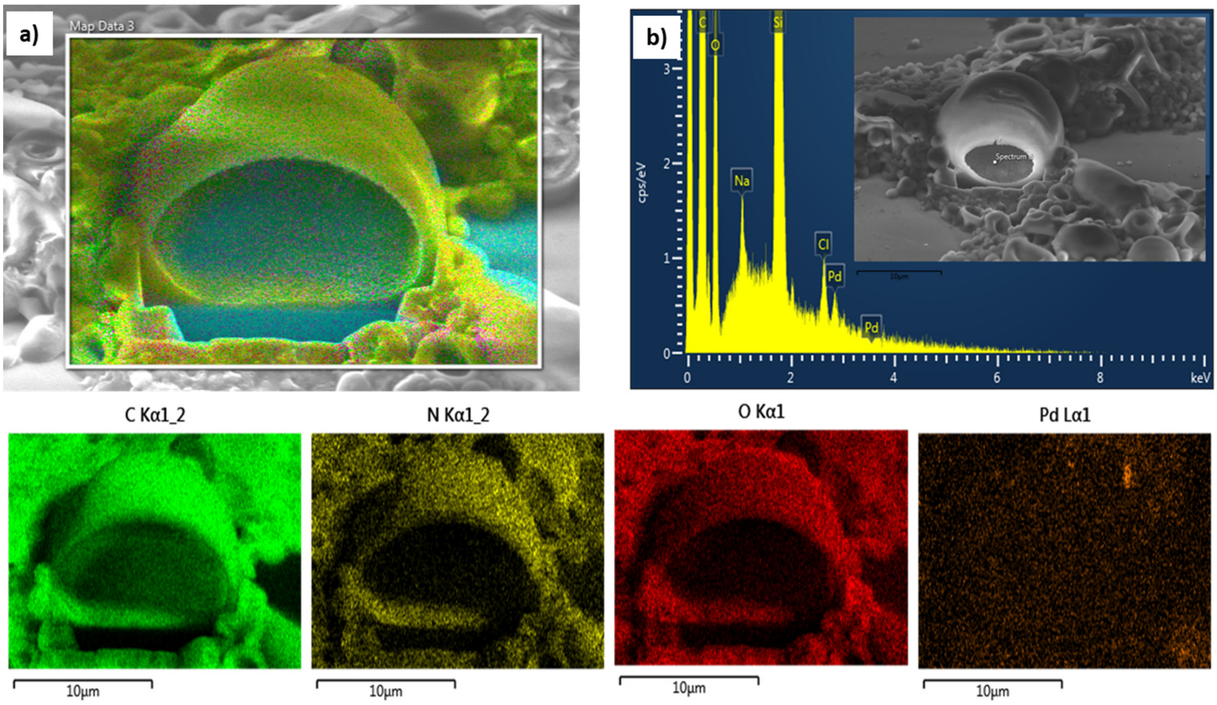

Figure S4. FIB-SEM (a) Mapping and (b) EDXS analyses of a cut MC.

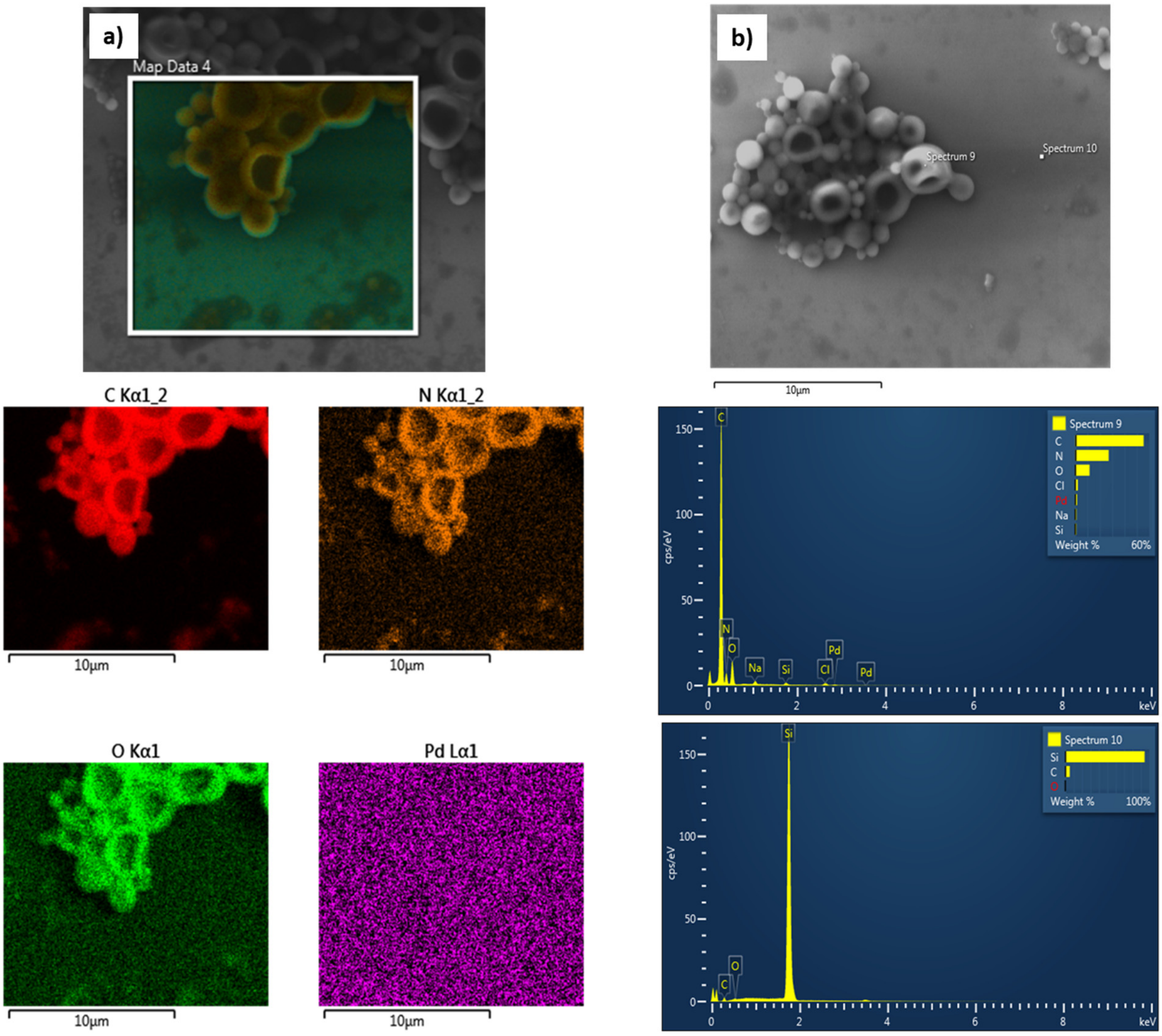

Figure S5. FIB-SEM applied for (a) mapping and (b) EDXS analyses of complete MCs of Pd_{NPs}/PEG_{200}@PU.

.
